# Effect of Pollen-Specific Sublingual Immunotherapy on Oral Allergy Syndrome: *An Observational Study*

**DOI:** 10.1097/WOX.0b013e3181752d1c

**Published:** 2008-05-15

**Authors:** Karl-Christian Bergmann, Hendrik Wolf, Jörg Schnitker

**Affiliations:** 1 Allergie-Centrum-Charité/ECARF, Department of Dermatology and Allergy, Charité-Universitätsmedizin Berlin, Berlin, Germany; 2 ALK-SCHERAX Arzneimittel GmbH, Wedel, Germany; 3 Institut für angewandte Statistik GmbH, Bielefeld, Germany

**Keywords:** immunotherapy, oral allergy syndrome, pollen, rhinoconjunctivitis, sublingual

## Abstract

**Background:**

Oral allergy syndrome (OAS) triggered by fruit and vegetables often occurs in patients with pollen-induced rhinoconjunctivitis because of cross-reactive epitopes in pollen and associated foods. This open observational study examined the effect of pollen-specific sublingual immunotherapy ([SLIT] B. U. Pangramin or SLITone involving birch/alder/hazel, grasses/rye, and/or mugwort) on OAS triggered by several foods in patients treated in standard practice. Very few studies have examined SLIT use in this situation.

**Methods:**

Patients (n = 102) had pollen-induced rhinoconjunctivitis and OAS and were followed for up to 12 months. Baseline OAS (triggers, symptoms, and symptom severity) was assessed by questionnaire and patient history. Change in OAS was assessed using oral challenge test with 1 or 2 dominant food triggers (and compared with the sum score calculated from the OAS questionnaire at baseline) and clinician ratings of change. Pollen-induced rhinoconjunctivitis symptoms and medication use were also measured.

**Results:**

In the oral challenge test, 77.0% of patients were considered responders (decrease in sum score of ≥ 50%; no difference in patients receiving B. U. Pangramin or SLITone). At baseline, investigators rated OAS severity as at least moderate in 94.9% of patients compared with 36.9% after 12 months of treatment. After 12 months, OAS was rated as much or very much improved in 72.9% of patients. Sublingual immunotherapy significantly reduced rhinoconjunctivitis symptoms and medication use. Only 10% of patients experienced adverse drug reactions.

**Conclusion:**

This study supplements the sparse literature on this topic and suggests that pollen-specific SLIT can reduce OAS triggered by pollen-associated foods in patients with pollen-induced rhinoconjunctivitis.

## 

Up to 70% of patients with pollen allergy also experience oral reactions to food (oral allergy syndrome [OAS]), typically fruit and vegetables [1-3]. Although most cases of OAS result in mild symptoms (itching of lips, mouth, and throat), more severe (lip and tongue swelling, angioedema of the pharyngeal mucosa) and even life-threatening (anaphylactic) symptoms can occur [4,5].

Specific immunotherapy with pollen allergens may have a beneficial effect on pollen-associated food allergy triggering OAS because of cross-reactive epitopes present in pollen and associated foods [6,7]. However, studies investigating the effect of subcutaneous pollen-specific immunotherapy on OAS report mixed results: some authors describe a reduction in OAS, whereas others have found no or only limited benefits from immunotherapy [8-14].

Pollen-specific sublingual immunotherapy (SLIT) is an alternative way of administering specific immunotherapy, which has been shown to be effective in reducing pollen-induced allergic rhinitis in several studies [15-19]. Sublingual immunotherapy is applied directly to the oral mucosa, the site of the OAS reaction, and may therefore offer additional benefits over subcutaneous immunotherapy. This proposal is consistent with general clinical experience that initial mild adverse reactions to SLIT, such as oral itching and mucosal swelling, typically disappear during continued application of SLIT [20].

To our knowledge, only 2 studies have investigated the effects of pollen-specific SLIT on OAS triggered by pollen-associated foods [21,22]. Hansen et al[21] compared SLIT and subcutaneous immunotherapy with placebo in birch-allergic patients with OAS triggered by apple. No effect of immunotherapy on OAS was found, as assessed by a questionnaire and an open oral challenge test, but the number of patients receiving SLIT was small (n = 11). Kinaciyan et al[22] also failed to find an effect of birch-pollen SLIT on OAS to apple. Recently, however, Enrique et al[23] showed that SLIT was effective in reducing food allergies: hazelnut-intolerant patients (n = 12) demonstrated significant increases in tolerance to hazelnut after SLIT with hazelnut extract as assessed by a double-blind placebo-controlled food challenge in a randomized, double-blind, placebo-controlled study.

This open observational study further explores the influence of SLIT on OAS, thereby adding to the very limited literature in this area. The effects of immunotherapy with various pollen allergens in patients with pollen-induced rhinoconjunctivitis and pollen-associated OAS were recorded by practicing allergists during routine application of commercially available SLIT products.

## Methods

### Patients

Patients had pollen-induced allergic rhinoconjunctivitis and concomitant OAS and did not have known contra-indications to specific immunotherapy. Data were recorded between September 2002 and June 2005 during routine SLIT treatment by practicing allergists located throughout Germany.

### Study Design

The study was open, noninterventional, and observational. Sublingual immunotherapy was administered by patients at home. Before SLIT initiation, demographic data, the history, and diagnostic measures with respect to pollen allergy and OAS were recorded. In an OAS questionnaire completed by the patients at that time, patients were asked to specify all foods that triggered their OAS symptoms, to name 1 or 2 foods provoking the most severe symptoms, to record the severity of symptoms (none/mild/moderate/severe) when last exposed to these foods, and to record the time of OAS onset after food intake. A weighted sum score was calculated from the 18 different symptoms assessed in this questionnaire (Table 1). Additional assessments (OAS severity, change in OAS status, response to foods) were performed during patient visits at the end of the initial treatment phase, and after 6 and 12 months of maintenance treatment (Table 2). The OAS severity and change in OAS status were assessed by the physician using the 7-point Clinical Global Impression Scales (OAS severity: not ill/borderline case/mildly ill/moderately ill/markedly ill/severely ill/among the most extremely ill; change of OAS status: very much improved/much improved/only minimally improved/unchanged/minimally worse/much worse/very much worse). Response to foods was rated as: food avoided/food tolerated without reaction/food better tolerated/reaction to food unchanged/food tolerance deteriorated. An open oral challenge test with the 1 or 2 foods identified as the most severe OAS triggers was scheduled after 6 and 12 months of maintenance therapy. The severity of symptoms triggered by the food challenge were assessed using a scale of none/mild/moderate/severe for 18 different symptoms and evaluated as weighted sum score as used for the OAS questionnaire (Table 1). Results from this test were compared with the weighted sum scores calculated from the OAS questionnaire at the beginning of SLIT.

**Table 1:** The OAS Questionnaire and Weighted Sum Score

**No**.	Symptom	Weight
1	Itchy lips	1
2	Itchy tongue	1
3	Itchy oral mucosa	1
4	Burning tongue	1
5	Swelling of the lips	1
6	Swelling of the tongue	1
7	Swelling of the oral mucosa	1
8	Swelling of the larynx	1
9	Inflammation of the tongue	1
10	Inflammation of the oral mucosa	1
11	Eye symptoms	2
12	Nose symptoms	2
13	Perioral skin symptoms	2
14	Wheeze	2
15	Dyspnea	3
16	Nausea/vomiting	3
17	Gastrointestinal disorders	3
18	Collapse, shock	4

**Table 2:** Summary of Assessments During the Study

	Study Start	Initial Treatment	Maintenance Treatment	
			
			6 Mo	12 Mo
Demographics and history of pollen allergy	•			
History and current diagnosis of OAS	•			
OAS questionnaire	•			
Severity of OAS	•	•	•	•
Course and global improvement of OAS		•	•	•
OAS challenge test (1 or 2 times)			•	•
Rhinoconjunctivitis symptoms	•*		•**	•**
Rhinoconjunctivitis medication	•*		•**	•**
Nasal provocation test	•		•	•
SLIT, SLIT safety		•	•	•
Patients' diary			•	•

*Last pollen season.

**Once after first SLIT season.

$$\text{Weighted sum score }=\overset{18}{\underset{\text{i}=1}{\sum }}\text{weigh}{\text{t}}_{\text{i}}\times \text{sympto}{\text{m}}_{\text{i}}$$

A rhinoconjunctivitis symptom score from 0 to 21 was obtained by totaling scores for 3 eye symptoms (itching/tear flow/and redness) and 4 nasal symptoms (obstruction/itching/rhinorrhea/sneezing): each symptom was scored retrospectively for the preceding pollen season on a scale of 0 to 3 (no/mild/moderate/severe symptoms). Lung symptoms were assessed for 4 items (chest tightness/wheezing/cough/dyspnea), and skin symptoms, for 2 items (pruritus/eczema). Optionally, a nasal provocation test (NPT) was performed according to the Position Paper of the German Society for Allergology and Clinical Immunology about application of the NPT on diseases of the upper airways [24].

Patients were also asked about medication use (Yes/No for a range of typical allergy medications) and whether sleeping was impaired by their allergy symptoms. Rhinoconjunctivitis symptoms were reassessed during the first pollen season after starting SLIT, and the NPT was optionally performed. At the end of the 12-month observation period, the status of the patient in the first pollen season with SLIT compared with the pretreatment season was assessed globally by patient and physician (better/unchanged/worse/not assessable). The doses applied and potential adverse reactions throughout the study were recorded in patient diaries.

### SLIT Preparations and Dosage

Sublingual immunotherapy was typically initiated with B. U. Pangramin SLIT pollen allergens [grass mix/rye, tree mix (hazel/alder/birch) and mugwort; ALK-SCHERAX Arzneimittel GmbH, Wedel, Germany], although a few patients started on SLITone (available from November 2003; ALK-SCHERAX Arzneimittel GmbH). The recommended treatment regimen for B. U. Pangramin SLIT involved an initial 4-week updosing phase followed by maintenance treatment involving 10 drops from a vial of strength 500 Specific Treatment Units Abello (STU)/mL 3 times a week. For SLITone, the recommended regimen involved an initial 10-day updosing phase followed by a single daily maintenance dose of 200 STU (0.2 mL of 1000 STU/mL concentration). During the updosing phases, patients were advised to increase the dose according to the dosage schedule only if the previous dose had been applied and if the dose was well tolerated. If adverse reactions occurred, patients were instructed to contact their physician. For both products, patients were told to keep the SLIT drops under the tongue for 2 to 3 minutes before swallowing. During the study, some patients switched from B. U. Pangramin SLIT to SLITone grass mix and rye or tree mix. In these cases, treatment with SLITone was always started with the initial 10-day updosing phase.

## Results

### Patient Characteristics

Overall, 102 patients were treated with at least 1 dose of SLIT (88 patients were treated with B. U. Pangramin SLIT, 11 were initially treated with B. U. Pangramin and then transferred to SLITone; 3 patients initiated SLIT with SLITone). Seventy-seven patients were considered to have completed the 12-month observation period. Reasons for not completing the study were adverse events (n = 8), lack of compliance (patients, n = 6; investigators, n = 3), improvement (n = 3), lack of efficacy (n = 2), lack of compliance + lack of efficacy (n = 1), termination by the patient (n = 1), and desire to start a family (n = 1); data from 2 of these patients were, however, included in some of the analyses. Of the 99 patients receiving B. U. Pangramin SLIT, 87 achieved the maximum maintenance dose after 4 weeks (n = 75) or delayed after a longer updosing phase (n = 12). Three patients were lost to follow-up at an unknown maintenance dose, 4 discontinued treatment at a submaximum level because of allergic adverse reactions, 3 patients named allergic symptoms because of pollen exposure as the reason for discontinuing updosing (no subsequent continuation was documented), and in 2 patients, a submaximum maintenance dose was chosen because of other medical reasons. In total, the maximum B. U. Pangramin SLIT dosage was reached in 87.9% of the patients.

Patient characteristics and OAS history at baseline are shown in Table 3. Patients were most frequently sensitized to birch/alder/hazel followed by grasses/rye and mugwort. Accordingly, patients experienced OAS reactions predominantly to tree-associated foods: apple (81.8%) and hazelnut (73.7%), and with lower frequency to carrot (27.3%). Patients completing the OAS questionnaire named 175 dominant triggers as causing the most marked symptoms. The most commonly named dominant triggers in the questionnaire were apple (33.1%, 58/175), hazelnut (22.3%, 39/175), carrot (8.0%, 14/175), peach (3.4%, 6/175), and celery (3.4%, 6/175). Symptoms to these dominant triggers mostly occurred within 5 minutes of eating the food, and 25.4% of symptoms were considered severe. Only 7.1% of reactions required immediate medical attention. Most patients (79.4%) received SLIT with tree allergens, 37.3% received SLIT with grasses and rye, and 5.9% received SLIT with mugwort.

**Table 3:** Patient Characteristics and OAS History at Baseline

Patients Treated With SLIT	n = 102
Median age, yrs (range)	34.5 (6-64)
Children < 14 yrs, %	13.7
Male/female, %	33.3/66.7
Median duration of rhinoconjunctivitis, yrs (range)	8.0 (1-37)
Patients with pollen-induced airway symptoms, %	62.7
Patients with pollen-induced skin symptoms, %	23.5
Positive skin prick test to:	n (%)
Grasses and rye	63 (67.7)
Birch, alder, hazel	93 (93.9)
Beech, oak, ash	35 (50.7)
Mugwort	26 (34.2)
Other pollen	5 (12.8)
House dust mites	27 (34.2)
Animal dander	22 (31.0)
SLIT treatment with (includes multiple entries):	n (%)
Tree mix (birch, alder, hazel)	81 (79.4)
Grasses and rye	38 (37.3)
Mugwort	6 (5.9)
OAS history	
Patients evaluable	n = 99
Median duration of OAS, yrs (range)	5.0 (0-23)
Predominant triggers, %	
Apple	81.8
Hazelnut	73.7
Carrot	27.3
Predominant symptoms	OAS reactions with this symptom, %
Itchy lips, tongue, and/or oral mucosa	83.4
Swelling of lips, tongue, oral mucosa, and/or larynx	74.3
Burning of tongue	40.0
Eye symptoms	34.9
Nose symptoms	32.6

### Effect of SLIT With Pollen Allergens on OAS

**Figure 1:**
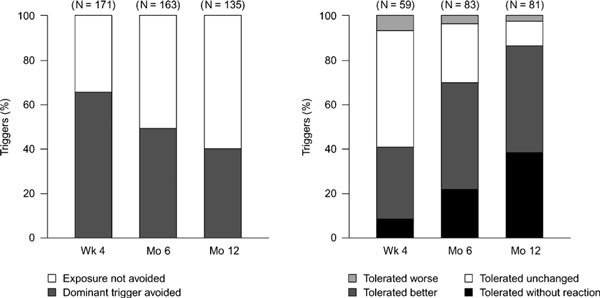
**Exposure to, and tolerability of, dominant OAS triggers**.

During the course of the study, patients frequently avoided foods that provoked OAS reactions. The rate of food avoidance for dominant OAS triggers decreased from 65.5% after 4 weeks' treatment to 40.0% after 12 months (Figure 1); 71 (40.6%) of the 175 named dominant triggers of OAS were avoided during the study. After 4 weeks' treatment, 24 (40.7% of patient cases) of the 59 food types ingested were tolerated without reaction or better than previously. After 12 months, this value increased to 86.4% (70/81).

**Figure 2:**
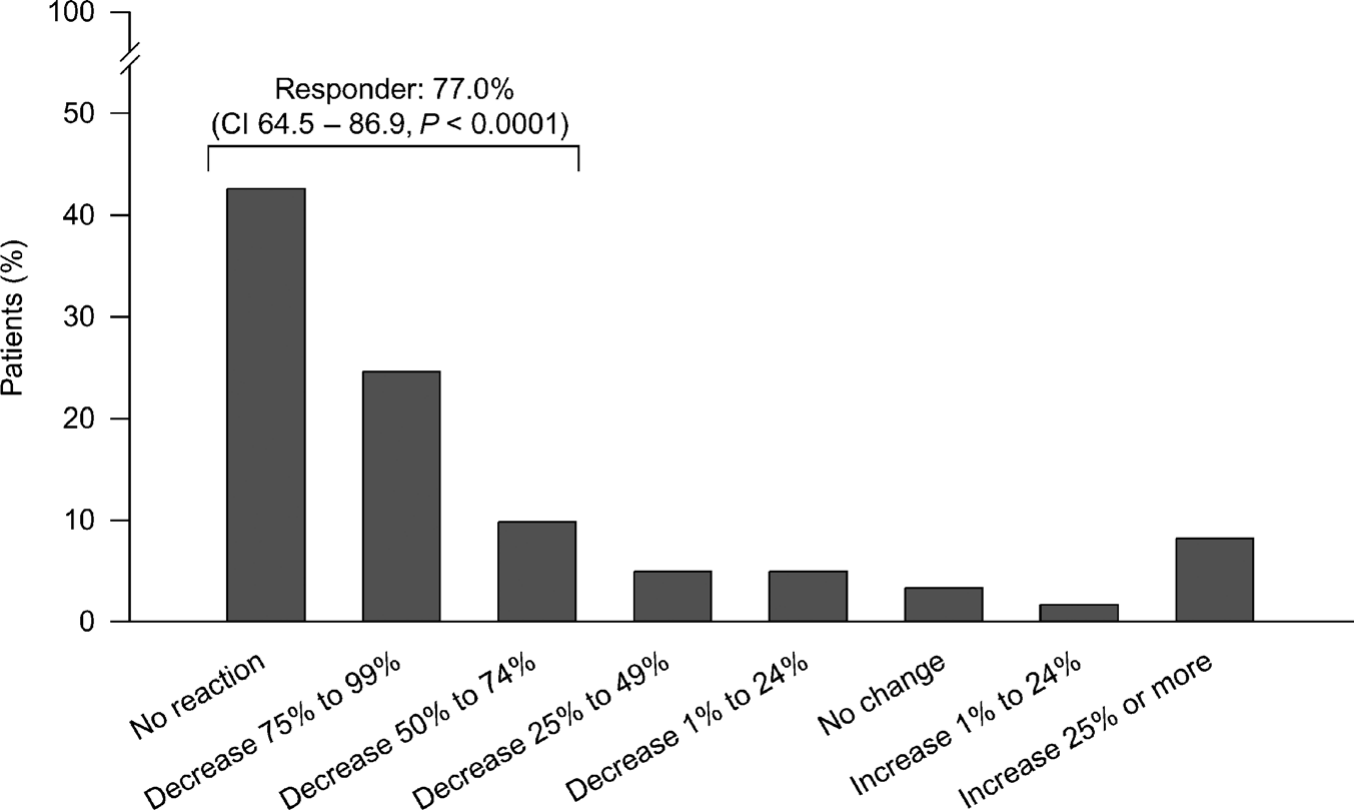
**The OAS challenge test at 6 and 12 months compared with the results of the OAS questionnaire at baseline (weighted sum score)**. The figure presents individually the last findings for each included patient (n = 61; 8 tests at 6 months, 53 tests at 12 months). CI indicates confidence interval.

In the oral challenge test, weighted sum score decreased by a mean of 64.3 relative to the reactions recorded in the OAS questionnaire before SLIT initiation. Defining a response as a decrease in sum score of 50% or more, 77.0% of patients were responders in the oral challenge test (confidence interval, 64.5-86.9, *P *< 0.0001, n = 61), and most of these patients experienced a reduction in sum score of more than 75% (Figure 2). There was no significant difference in response in patients receiving B. U. Pangramin SLIT or SLITone. In patients with apple intolerance (the most common food trigger) who underwent an oral challenge test with this food, 89.2% (33/37) responded to SLIT (decrease in sum score of at least 50%). The respective percentage for hazelnut-intolerant patients was 69.6% (16/23).

Investigators reported a decrease in OAS severity after SLIT; at baseline, 94.9% of patients were rated as at least moderately ill compared with 36.9% at month 12. Furthermore, after 6 months of SLIT treatment, investigators rated OAS as much or very much improved in 53.9% (48/89) of patients. This percentage increased to 72.9% (54/74) in patients completing 12 months of therapy. In all patients receiving treatment (regardless of how long), 65.7% (65/99) were rated by the investigators as much or very much improved.

### Effect of SLIT on Pollen-Induced Rhinoconjunctivitis

**Figure 3:**
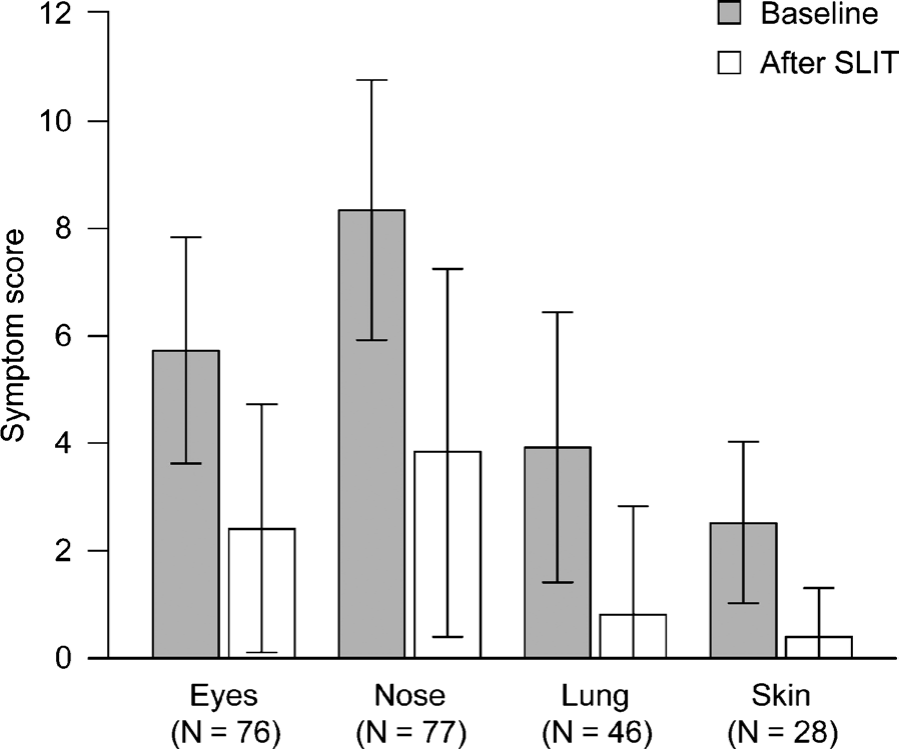
**Course of rhinoconjunctivitis symptom scores**.

Sublingual immunotherapy treatment markedly decreased rhinoconjunctivitis symptoms during the pollen season in the completer population (Figure 3). The mean symptom severity score for eye symptoms decreased by 58.4%, nose symptoms by 54.2%, bronchial symptoms by 79.2%, and skin symptoms by 85.9%. Insomnia improved in 60.0% of cases.

After SLIT, symptomatic medication was reduced or no longer required in 92.1% (n = 70) of patients who were using medication at baseline (n = 76). Forty-nine patients completed an NPT before and after SLIT; 34.7% of them had a positive test before and a negative test after SLIT. Of patients and investigators, 84.2% rated well-being during the pollen season as improved after SLIT relative to the last pollen season before immunotherapy. There was no difference in well-being assessment between B. U. Pangramin and SLITone.

### Tolerability of SLIT

Of the 99 patients who received at least 1 dose of B. U. Pangramin SLIT, 10 (10.1%) had a total of 14 adverse drug reactions [asthma attack (n = 2), nausea (n = 2), retching, neurodermatitis, angina, diarrhea, OAS worsening, anaphylactic event of undetermined origin, prickling of the tongue (n = 2), numbness of the tongue, burning of the tongue]. No adverse drug reactions were recorded with SLITone. Only 1 patient discontinued SLIT because of deterioration of OAS after the initial therapy. In total, 8 of the adverse drug reactions resulted in treatment discontinuation. These included asthma (n = 2) and single cases of OAS worsening, severe angina, neurodermatitis, nausea, anaphylactic event of undetermined origin (not temporally related to SLIT), and prickling tongue. Overall, 94.7% of patients and 95.7% of physicians rated the tolerability of initial SLIT as very good (patients, 76.8%; physicians, 77.7%) or good (patients, 17.9%; physicians, 18.1%). Similar results were reported after 6 and 12 months of maintenance therapy (patients, 93.9%; physicians, 96.3%; and patients, 94.7%; physicians 96.1%, respectively).

## Discussion

In this open observational study, we found that patients treated with pollen-specific SLIT during routine clinical treatment showed improvements in OAS triggered by various pollen-associated food allergens (when these foods were ingested naturally or during an oral challenge test) and in pollen-induced rhinoconjunctivitis. Furthermore, the improvement increased as treatment continued for up to 12 months. Physicians rated OAS as much or very much improved in nearly 75% of patients after 12 months of treatment, and 77% of patients had a favorable response in the open oral challenge test. The symptoms of allergic rhinoconjunctivitis were also improved during the first pollen season after SLIT treatment.

Although our study was not controlled, OAS does not normally improve without treatment [2,8,11]. For example, in a study of specific injection immunotherapy in birch pollen-sensitive patients with apple-induced OAS, none of the 20 control patients reported a reduction in symptoms during a 12- to 48-month follow-up period[8]; in contrast, 84% of immunotherapy-treated patients reported complete disappearance or significant improvement in symptoms. It is our opinion that an improvement in OAS symptoms to the extent reported in this study would not normally be expected without treatment. Furthermore, annual variations in pollen count are unlikely to account for our findings because patients were recruited and treated over several years and were allergic to different pollens.

To our knowledge, only 2 other studies[21,22] have investigated the effect of pollen-specific SLIT in patients with concomitant OAS. In both studies, no effect of birch-allergen SLIT or subcutaneous immunotherapy on OAS triggered by apple was reported relative to placebo, but the number of patients treated with SLIT and assessed by food challenge was low (11 patients[21] and 9 patients[22]). The recently published study by Kinaciyan[22] classified patients allergic to birch who had been treated with SLIT as responders and nonresponders according to the outcome in an NPT. The 9 patients who had a positive response in the NPT were tested by double-blind placebo-controlled food challenge and oral food challenge with apple. No significant differences between the food challenges at baseline and after 1 year of SLIT were observed. The authors speculate based on the results of T-cell proliferation experiments that an exclusively food-specific T-cell response to Mal d 1 (a protein with an amino acid sequence that is 64% similar to the major birch pollen allergen, Bet v 1) may exist that can only be altered if OAS-related food allergens are contained in the vaccine for SLIT. Consistent with this argument, another study showed that patients with OAS and food allergy to hazelnut can be successfully treated with SLIT using a hazelnut extract containing the most relevant allergens triggering OAS and food allergy [23].

Nevertheless, our observational study involving a much larger sample of 102 patients with pollen-induced allergy and concomitant OAS suggests that patients treated routinely with pollen allergen SLIT can report positive effects of SLIT on OAS. This is consistent with several studies that have shown benefits of subcutaneous pollen-specific immunotherapy on concurrent OAS using open[2,8,13,14] or blinded, [11] and controlled[2,8,11,14] or uncontrolled[13] designs. Furthermore, SLIT may have the potential to influence OAS more strongly than subcutaneous treatment because of the application of allergens directly to the oral mucosa and the subsequent induction of oral tolerance. To explore the effect of SLIT on OAS further, controlled blinded clinical trials should be undertaken. Nevertheless, this study extends the available literature in this field, particularly by studying a wider variety of patients, treatments, and OAS triggers, more indicative of the real-life clinical setting.
